# Synthesis of iridium-based nanocomposite with catalase activity for cancer phototherapy

**DOI:** 10.1186/s12951-021-00948-8

**Published:** 2021-07-07

**Authors:** Hang Wu, Qi Jiang, Keyi Luo, Chunping Zhu, Mengmeng Xie, Shige Wang, Zhewei Fei, Jiulong Zhao

**Affiliations:** 1 Department of Breast Surgery, Xinhua Hospital, Shanghai Jiaotong University School of Medicine, No. 1665 Kongjiang Road, Shanghai, 200433 People’s Republic of China; 2 Department of Gastroenterology, Changhai Hospital, Second Military Medical University, No. 168 Changhai Road, Shanghai, 200433 People’s Republic of China; 3 College of Science, University of Shanghai for Science and Technology, No. 334 Jungong Road, Shanghai, 200093 People’s Republic of China

**Keywords:** Photothermal therapy, Photodynamic therapy, Cancer treatment, Biocompatibility, Iridium nanocomposite

## Abstract

The combination of photothermal therapy (PTT) and photodynamic therapy (PDT) has attracted attention due to its enhanced tumor therapy effect. This study proposes a novel nanoenzyme-based theranostic nanoplatform, IrO_2_@MSN@PDA-BSA(Ce6), for the combined PTT and PDT of tumors. IrO_2_ was prepared by a simple hydrolysis method and coated with a thin layer of mesoporous silica (MSN) to facilitate the physical adsorption of Chlorin e6 (Ce6). The PDA coating and IrO_2_ NPs of the nanoplatform demonstrated an improved photothermal conversion efficiency of 29.8% under NIR irradiation. Further, the Ce6 loading imparts materials with the ability to produce reactive oxygen species (ROS) under 660 nm NIR laser irradiation. It was also proved that the IrO_2_ NPs could catalyze the hydrogen peroxide (H_2_O_2_) in the tumor microenvironment (TME) to generate endogenous oxygen (O_2_), thereby enhancing the efficiency of PDT. The in vitro and in vivo experiments indicated that the nanocomposite was highly biocompatible and could produce a satisfactory tumor therapeutic effect. Thus, the findings of the present study demonstrate the viability of using theranostic nanoenzymes for translational medicine.

**Supplementary Information:**

The online version contains supplementary material available at 10.1186/s12951-021-00948-8.

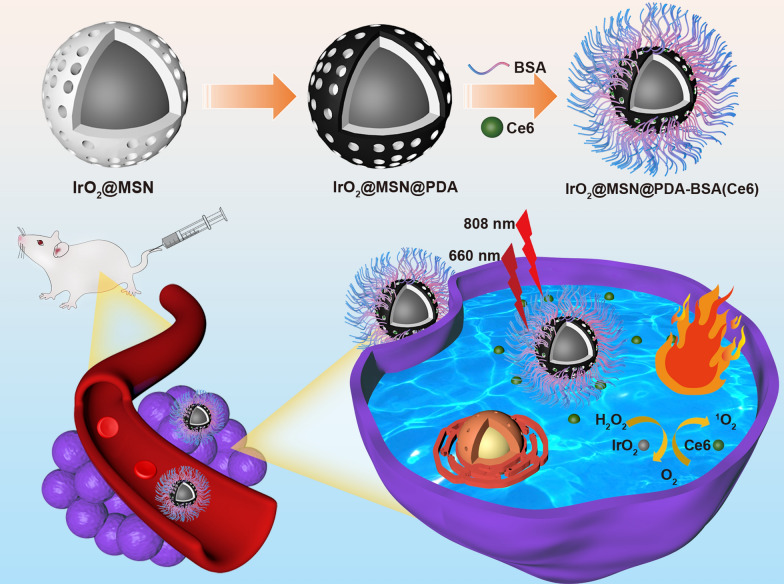

## Introduction

Cancer is currently one of the major obstacles preventing the reduction of the global mortality rate [1, 2]. Traditional cancer treatment strategies, such as chemotherapy, radiation therapy, and surgery, result in unavoidable side effects, the development of drug resistance, and ineligibility for surgery [3–5]. The limitations of these treatments have motivated researchers to develop new cancer treatments with relatively few side effects and a high efficiency. Near-infrared light (NIR)-induced tumor therapies, such as photothermal therapy (PTT) and photodynamic therapy (PDT), have attracted the attention of many researchers because they are highly effective, non-invasive, spatiotemporally controllable, and lead to relatively few side effects [6, 7].

PDT involves the absorption of photon energy by a photosensitizing agent (PSA), resulting in the transfer of its electrons to the oxygen molecules (O_2_) in the cancer cells. This leads to the production of a type of highly toxic reactive oxygen species (ROS, e.g., ^1^O_2_) that cause irreparable damage to the cancer cells [8]. A light operative dose, reasonable PSA concentration, and adequate oxygen are necessary for effective tumor ablation. In addition, the amount of O_2_ directly affects the efficiency of PDT [9]. Due to the rapid and uncontrolled proliferation of tumor cells, the O_2_ levels in solid tumors are inadequate, thereby reducing the therapeutic efficiency of PDT [10, 11]. Thus, researchers have proposed innovative strategies to enhance the O_2_ concentration in tumors [12]. Oxygen carriers, such as perfluorocarbon (PFC) nanoparticles NPs, can facilitate continuous oxygen supply and have been used to enhance the O_2_ concentration in tumors for PDT [13]. Enzyme-like substances with catalase activity have recently been used with PSA to counter hypoxia and enable ROS generation [14]. Although the H_2_O_2_ level in tumor microenvironment (TME) is relatively high compared to the normal tissues, the amount of endogenous H_2_O_2_ is still insufficient to kill the cancer cells due to the cancer cells characteristically have a high antioxidant capacity [15]. Researchers have discovered that the overexpression of H_2_O_2_ in TME can be utilized for the catalytic generation of endogenous O_2_ and facilitate tumor PDT [16, 17]. Nanoenzymes, in comparison with natural catalase, have a relatively low cost, high activity, and qualified thermostability [18, 19]. Therefore, they can be employed to enhance the therapeutic effect of PDT.

PTT is an alternative phototherapy technique that can utilize the photothermal effects of photothermal transduction agents (PTAs) to raise the temperature of the surrounding environment and trigger the ablation and apoptosis of cancer cells [20, 21]. The application of a PTA with high biocompatibility and efficient photothermal conversion efficiency is likely to improve the efficiency of photothermal therapy [22]. Several PTAs, such as two-dimensional (2D) materials [23], noble metal materials [24], metal chalcogenide materials [25], and conjugated polymers (e.g., polydopamine (PDA) and polypyrrole (PPy)), have been synthesized in recent years [26, 27]. However, owing to the poor performance of a single PTA, high-energy NIR laser irradiation or a relatively strong dose of PTA is required to obtain the desired treatment effect [28]. In addition, it is difficult to achieve satisfactory therapeutic activity by solely using PTT [29]. Thus, modern studies are focusing on dual-mode therapy combinations of PTT and PDT [30, 31]. The main obstacle involves building a reasonable nanoplatform to maximize the combined effects of PTT and PDT to kill tumor cells.

The application of iridium oxide (IrO_2_) has recently drawn attention due to its high biocompatibility and photothermal conversion efficiency [32]. Studies have discovered that IrO_2_ has catalase (CAT)-like activity that enables it to catalyze H_2_O_2_ in the TME to generate endogenous O_2_, thereby enhancing the efficiency of PDT [33]. However, few studies have utilized IrO_2_ nanomaterial-based nanoplatform systems for the combined PTT and PDT treatment of tumors. An IrO_2_@MSN@PDA-BSA nanocomposite was synthesized in this study for the PTT and PDT dual-mode therapeutic treatment of tumors. IrO_2_ was prepared by a simple hydrolysis method and coated with a thin layer of mesoporous silica (MSN) to facilitate the physical adsorption of Chlorin e6 (Ce6). The MSN layer can not only protect the oxidation of IrO_2_ but also provide abundant functional groups to support the further surface modification of the nanocomposite. Subsequently, PDA was coated on the surface of IrO_2_@MSN, followed by the grafting of bovine serum albumin (BSA) on the surface of IrO_2_@MSN@PDA as a stabilizer. The installation of IrO_2_@MSN@PDA-BSA (Ce6) serves several purposes. The PDA coating and IrO_2_ NPs demonstrate significant photothermal conversion under NIR irradiation. IrO_2_@MSN@PDA-BSA(Ce6) can produce ROS to kill cancer cells under photon activation. IrO_2_ can also catalyze the decomposition of H_2_O_2_ to enhance the production of O_2_ in the TME, thereby enhancing the therapeutic effect of PDT. In vitro and in vivo experiments have proved that IrO_2_@MSN@PDA-BSA(Ce6) is biocompatible and can passively target tumors through the enhanced permeability and retention (EPR) effect, thereby harnessing the clinical and combined effects of PTT and PDT for the tumors. The design of IrO_2_@MSN@PDA-BSA(Ce6) provides a framework for the design of multifunctional nanocomposite that can accurately treat cancer.

## Experimental section

### Preparation and characterization of IrO_2_@MSN@PDA-BSA and the loading of Ce6

#### Synthesis of IrO_2_-PVP NPs

For the synthesis of IrO_2_-PVP nanoparticles, first, 0.05 g IrCl_3_ and 0.1 g PVP were dissolved in 15 mL of distilled water by magnetic stirring (400 rpm) at room temperature. Then, the solution pH was adjusted to 12 using NaOH solution (2.0 M), and the mixture was allowed to react at 80° C for 12 h with stirring. Finally, the mixture was further separated by centrifugation (12,000 rpm, 5 min), and then rinsed three times with ethanol and distilled water. The sample in this experiment was freeze-dried and collected for the following usages.

#### Synthesis of IrO_2_@MSN NPs

For the synthesis of IrO_2_@MSN nanoparticles, the as-prepared IrO_2_-PVP NPs were dissolved in 65 mL ethanol upon ultrasonication for 30 min. Then, 2.8 mL NH_3_**·**H_2_O (28 %) was added dropwise and the mixture was stirred at room temperature for 30 min. Subsequently, 0.1 mL TEOS was dissolved in 6.5 mL ethanol and added dropwise to the mixture at a rate of 0.5 mL/min under vigorous stirring. After stirring at room temperature for 6 h, the obtained products were centrifuged and washed twice with ethanol and once with distilled water. To further remove the surfactant template of CTAB, the product was stirred with saturated ammonium nitrate ethanol solution for 12 h. The resulting product was centrifuged, and washed it with double-distilled water extensively.

#### Preparation of IrO_2_@MSN@PDA NPs

In this step, we dissolved 0.2 g dopamine in Tris buffer solution (40 mL, 0.01 M, pH = 8.5), and then mixed it with the as-prepared IrO_2_@MSN NPs. After stirring it at room temperature for 4 h, the color of the solution changed into dark brown because of the oxidation, and the formed PDA coated IrO_2_@MSN (IrO_2_@MSN@PDA) NPs were collected by centrifugation (8500 rpm, 5 min). The product was washed three times with ethanol and water to remove any possible remnants.

#### Preparation of IrO_2_@MSN@PDA-BSANPs

IrO_2_@MSN@PDA-BSA was synthesized by the amidation reaction between BSA and PDA. Specifically, the as-prepared IrO_2_@MSN@PDA and 0.25 g BSA were added to 15 mL phosphate buffer (Na_2_HPO_4_–NaH_2_PO_4_, pH = 8.0) under 4 h of ultrasounding at room temperature. Finally, the product was conventionally centrifuged (13,000 rpm, 10 min) and washed it three times with distilled water.

#### Ce6 loading of IrO_2_@MSN@PDA-BSA NPs

For the synthesis of IrO_2_@MSN@PDA-BSA(Ce6), Ce6 was dissolved in DMSO at a concentration of 1000 µg/mL. Afterwards, the prepared Ce6 stock solution was diluted into different concentrations and mixed with IrO_2_@MSN@PDA-BSA (IrO_2_@MSN@PDA-BSA finally at 1000 µg/mL, Ce6 finally at 50, 100, and 200 µg/mL). Then, the mixture was magnetically stirred overnight at room temperature. Thereafter, the reaction products were centrifuged at 12,000 rpm for 8 min to obtain IrO_2_@MSN@PDA-BSA(Ce6) NPs. The supernatant containing excess Ce6 was carefully collected for the loading amount calculation of Ce6. In brief, we used UV–vis–NIR spectroscopy to read the absorbance at 404 nm of the supernatant to reckon the concentration of Ce6 as per the absorbance-concentration standard curve. The loading efficiency of Ce6 was calculated by W_t_/W $\times$ 100 % (W_t_ and W stand for the weight of loading Ce6 and total Ce6, respectively.), and the loading percentage of Ce6 was calculated by W_t_/W_S_ $\times$ 100 % (W_t_ and W_s_ stand for the weight of loading Ce6 and the weight of IrO_2_@MSN@PDA-BSA, respectively.). The amount of released Ce6 was measured by using a UV–vis spectrometer. In detail, four groups of IrO_2_@MSN@PDA-BSA(Ce6) (1 mL, Ce6 = 42.9 µg/mL) were placed in a dialysis bag with a molecular weight cutoff of 14,000. Then, they were dialyzed in 4 mL of PBS solution (pH = 7.4) in a constant temperature oscillator at 37 °C) for 24 h. At each predetermined time point, 2 mL of buffer solution that contained the released Ce6 was taken out and 2 mL of the corresponding fresh buffer solution was added. The concentration of the released Ce6 was determined with UV–vis–NIR spectroscopy at 404 nm.

### Measurement of the dissolved oxygen content

In this step, the catalase-like catalytic efficiency of IrO_2_@MSN@PDA-BSA at different temperature (37 and 80 °C) was evaluated by mixing IrO_2_@MSN@PDA-BSA (finally concentration at 200 µg/mL) with various concentration of H_2_O_2_ (finally at 5, 10, 20 and 50 µM). After the addition of H_2_O_2_, we used an oxygen probe (JPBJ-608 portable dissolved oxygen meter, Shanghai REX Instrument Factory) to monitor the dissolved oxygen content, and use a digital camera to record the bubble releasing of the reaction. At the same condition, the IrO_2_@MSN@PDA without the addition of H_2_O_2_ was set as a control. The catalytic efficiency of IrO_2_@MSN@PDA-BSA under simulated TME (pH = 6.0, the concentration of H_2_O_2_ was about 500 µM) conditions was investigated. In details, IrO_2_@MSN@PDA-BSA (200 µg/mL) NPs were incubated in PBS solutions at different pH values (6.0 and 7.4) containing 500 µM H_2_O_2_. At predetermined time points, the generated dissolved O_2_ was measured by an oxygen probe (JPBJ-608 portable dissolved oxygen meter, Shanghai REX Instrument Factory).

### Detection of the singlet oxygen

1,3-diphenylisobenzofuran (DPBF), a classical probe for the measurement of singlet oxygen, was employed for the detection of the singlet oxygen. Briefly, 2.95 mL IrO_2_@MSN@PDA-BSA(Ce6) in dimethyl sulfoxide was mixed with 50 µL DPBF in dimethyl sulfoxide. The terminal concentration of IrO_2_@MSN@PDA-BSA(Ce6) and DPBF was 20 µg/mL and 10 µM, respectively. Then, the mixture was added with H_2_O_2_ (10 mM, 45 µL), while the mixture added with H_2_O (45 µL) was set as the control group. We exposed the above solutions to a 660 nm NIR laser (0.3 W/cm^2^) in a dark environment for 20 min, and used the UV–vis–NIR spectrophotometer (Lambda 25, PerkinElmer, USA) to record the absorption intensity at designed time intervals.

### Photothermal conversion performance

In order to explore the photothermal conversion performance, the as-prepared IrO_2_@MSN@PDA-BSA(Ce6) was prepared in different concentrations (0, 100, 250 and 500 µg/mL in saline). Then, 100 µL of the above samples were added in each well of a 96-well plate and exposed to the 808 nm NIR laser (1 W/cm^2^, 5 min). To investigate the power density-dependent thermal characteristics, IrO_2_@MSN@PDA-BSA(Ce6) with the concentration of 200 µg/mL was select to be irradiated with 808 nm NIR laser at different power (0.5, 0.8 and 1 W/cm^2^) for 5 min. The photothermal stability of IrO_2_@MSN@PDA-BSA(Ce6) was also validated via the 808 nm NIR laser irradiation (1 W/cm^2^) for 5 on/off cycles (laser on for 10 min and laser off for 10 min in each cycle). We use FLIR™ E60 camera (FLIR, USA) to record the temperature increment (ΔT) of the above experiment for specific analysis.

### In vitro cellular viability

In this section, L929 cells were chosen to assess the potential cellular cytotoxicity of IrO_2_@MSN@PDA-BSA in vitro. On the one hand, the CCK8 kit was performed to detect the cell viability after cultured with IrO_2_@MSN@PDA-BSA(Ce6). In brief, L929 cells were seeded into 96-well plate (8000 cells/well) and maintained at 37 °C in a humidified atmosphere with 5% CO_2_. After 24 h, the medium was replaced by various concentrations (100 µL, 0, 50, 100, 250 and 500 µg/mL in RMPI1640) of IrO_2_@MSN@PDA-BSA(Ce6). Another 24 h later, 100 µL fresh RMPI1640 with 10 µL CCK8 test solution was added in each tested well to replace the old solution and then incubated for another 2 h. Using a microplate reader to read the absorbency of each well at 450 nm to calculate cell viability. On the other hand, calcein-AM/PI Live/Dead kit was used to further determine the cytocompatibility of IrO_2_@MSN@PDA-BSA(Ce6). L929 cells were cultured and disposed of with the same as the CCK8 assay but lastly stained with calcein-AM/PI (100 µL) based on the manufacturer for 30 min at 37 °C. The counterstained cellular morphology was recorded using a fluorescence microscope (Olympus BX53).

### In vitro tumor therapy

To study the cytotoxic effects of IrO_2_@MSN@PDA-BSA(Ce6) in vitro, HT29 cells were seeded into 96-well plates at a density of 8 × 10^3^ cells/well and maintained in a humidified cell-incubator with 5% CO_2_ at 37 °C overnight for cell attachment. Then, each well was filled with 100 µL IrO_2_@MSN@PDA-BSA(Ce6) (in DMEM, 200 µg/mL) and continue culturing for 12 h. Then, we divided cells into four groups (n = 6). Cells in group 2 were irradiated with 808 nm laser (1 W/cm^2^, 5 min) to assess the effect of PTT. Cells in group 3 were exposed to 660 nm laser (0.3 W/cm^2^, 3 min) and cells in group 4 were treated with the medium containing H_2_O_2_ (100 µmol/L) and the pH was adjusted to 6.0 with HCl. Cells in group 5 were exposed to a combined NIR irradiation, including 808 nm (1 W/cm^2^) for 5 min and 660 nm (0.3 W/cm^2^) for 3 min. Cells treated with DMEM without other intervention were set as the control (group 1). Finally, we used CCK-8 and the calcein-AM/PI Live/Dead staining to evaluate the relative viabilities of cells.

### In vitro hemocompatibility

The hemocompatibility of IrO_2_@MSN@PDA-BSA(Ce6) was investigated by the typical in vitro hemolysis assay as below: fresh complete blood samples (1 mL) were collected from healthy KM mice and centrifuged (3000 rpm, 3 min, 4 °C) to obtain the mouse red blood cells (mRBCs). After that, the harvested mRBCs were washed with PBS three times and reposited in PBS for further experiments. Then, the above mRBCs dispersions (0.3 mL) was mixed with 1 mL of different concentrations (100, 250 and 500 µg/mL) of IrO_2_@MSN@PDA-BSA and cultured at 37 °C. Meanwhile, mRBCs dispersions (0.3 mL) were incubated with 1 mL of PBS (negative control) or 1 mL of DI water (positive control). After incubating for 4 h, the supernatant of the above mixture was gathered via centrifugation (12,000 rpm, 5 min). Subsequently, we recorded the absorbance of the supernatants at 580 nm to compute the hemolysis percentage (HP) according to literature.

### In vivo biocompatibility and biodistribution

To evaluate the biocompatibility and biodistribution of IrO_2_@MSN@PDA-BSA(Ce6) in vivo, the healthy KM female mice (SPF grade) were randomly sent into two groups (n = 24 per group). All animal experiments were conducted under the protocols approved by the Laboratory Animal Center of Changhai Hospital of Second Military Medical University, the policies of National Ministry of Hkiuealth. One group was intravenously (I.V.) injected with saline as a control, another group was injected with IrO_2_@MSN@PDA-BSA(Ce6) solution (250 µL, 1 mg/mL in PBS). The body weight of all experimental mice was weighed and recorded per 2 days. Mice were heart punctured for collect blood samples after anesthesia on 1, 7, 28 days and then were euthanized for organs collection. The blood samples were used for blood routine analysis and blood biochemical index testing in virtue of a Sysmex XS-800i automated hematology analyzer and a DxC 800 automatic biochemical analyzer. These crucial organs (heart, lung, liver, kidney, and spleen) were partly sectioned and immediately dipped in 4% polyformaldehyde for hematoxylin and eosin (H&E) staining. Besides, the remaining part of the organs was used for quantitative detection of the Silicon (Si) ion biodistribution through the Agilent 700 Series ICP-OES.

### In vivo tumor therapy

HT29 cells (10^7^ cells per mouse, in 150 µL of serum-free DMEM) were subcutaneously implanted into the right back of the Balb/c nude mice (female, 4–6 weeks old). Three weeks after the cell-injection, a tumor nodule about 0.15–0.20 cm^3^ in each mouse was established. Then, the tumor mice were randomly allocated to five groups with six mice in each group as follows: mice in group 1 were I.V. injected with saline and served as the control group. Mice in group 2 were given with IrO_2_@MSN@PDA-BSA(Ce6) solution (200 µL, 1 mg/mL in PBS, I.V.) for PTT. Mice in group 3 were treated with IrO_2_@MSN@PDA-BSA(Ce6) solution (200 µL, 1 mg/mL in PBS, I.V.) for PDT. With regard to the dual-modal tumor therapy, 200 µL (I.V.) and 20 µL (I.T.) IrO_2_@MSN@PDA-BSA(Ce6) were separately injected in group 4 and group 5. Twelve hours after the injection, mice in group 1 and group 2 were irradiated with 808 nm NIR laser (1.0 W/cm^2^) for 5 min, while mice in group 3 were subjected to the 660 nm laser (0.3 W/cm^2^) for 5 min. As the combined treatment group, the mice in group 4 and group 5 were successively exposed to 808 nm NIR laser (1.0 W/cm^2^) and 660 nm laser (0.3 W/cm^2^) for 5 min. The FLIR E60 infrared camera was exploited to keep the record of ΔT measurement and thermal imaging during the treatments. Mice in all groups were then raised for 4 weeks, and we measured the tumor volume every 3 days. The efficiency of in vivo tumor treatment was assessed by the relative tumor volume.

### Statistical analysis

All the data acquest from the above experiments were reported as mean ± standard deviation (SD) and statistically analyzed with one-way ANOVA. P values < 0.05 was regard as statistically significant. Data indicated with (*) deputize for p < 0.05, (**) for p < 0.01, and (***) for p < 0.001.

## Results and discussions

### Synthesis and characterization of IrO_2_@MSN@PDA-BSA

**Scheme 1:**
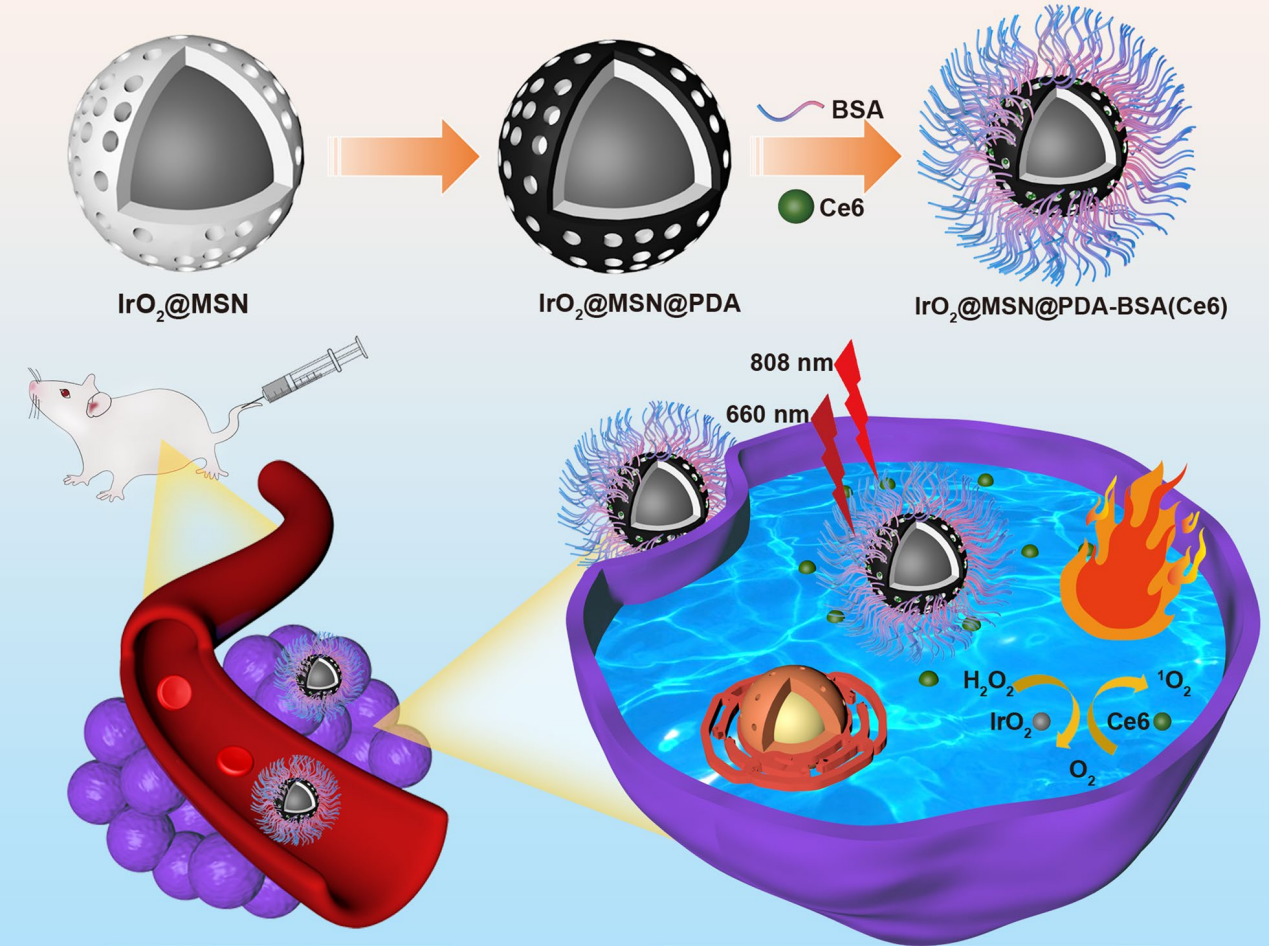
The schematic synthesis procedure and therapeutic mechanism of IrO_2_@MSN@PDA-BSA(Ce6)

**Fig. 1:**
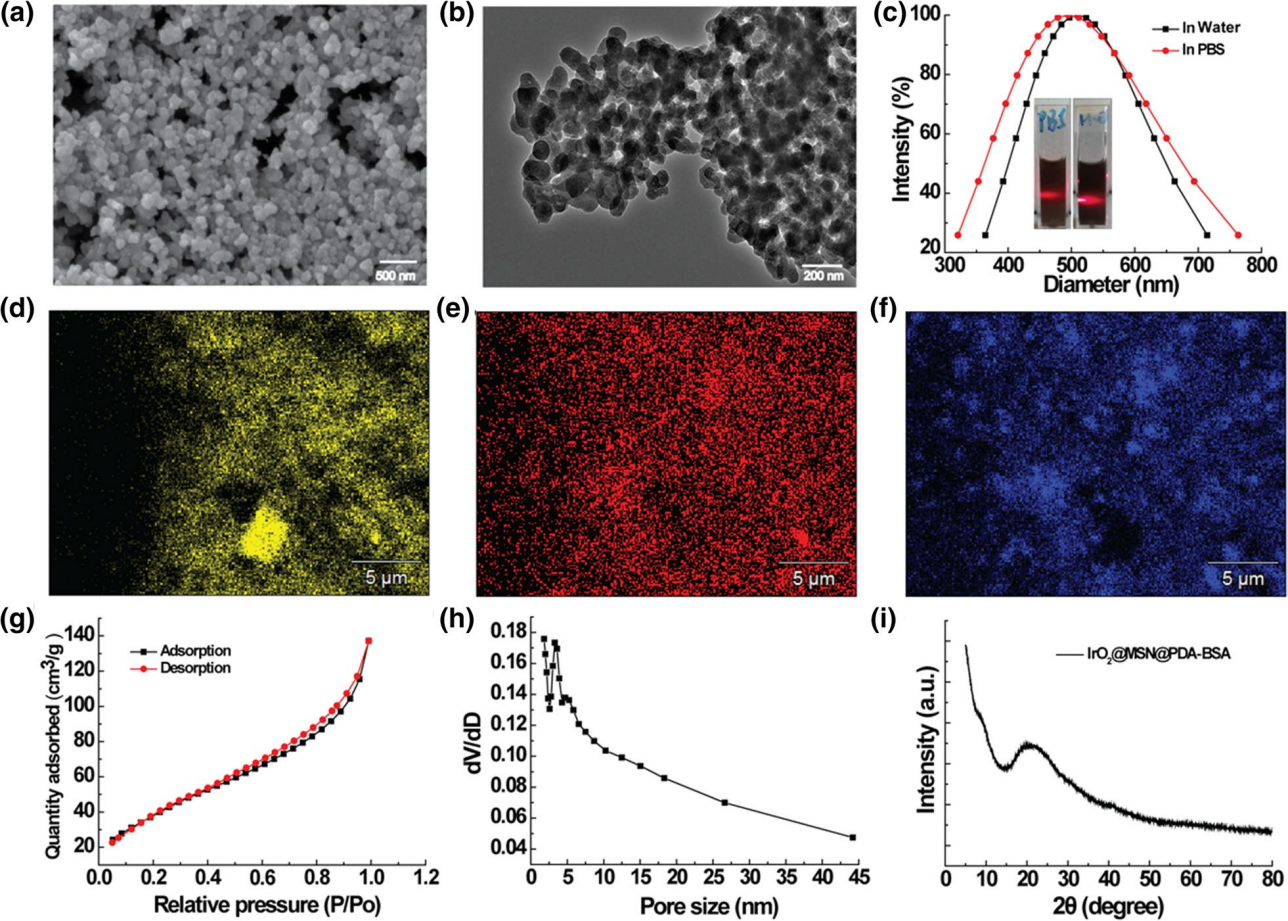
Characterization of IrO_2_@MSN@PDA-BSA NPs. **a** SEM; **b** TEM; **c** dynamic light scattering and solution photos (left: in PBS, right: in water); **d**–**f** elemental distribution mappings (d, O; e, Si; f, Ir); **g** nitrogen adsorption–desorption isotherm; **h** pore size of IrO_2_@MSN@PDA-BSA; **i** XRD pattern

Colloidal-stable polyvinylpyrrolidone (PVP)-decorated IrO_2_ NPs (IrO_2_-PVP) were prepared through facile hydrolysis of IrCl_3_, the as-prepared IrO_2_-PVP nanoparticles showed a diameter of approximately (10.12 ± 2.10) nm (Additional file 1: Fig. S1). Then the as-prepared nanoparticles were coated with a thin layer of MSN to carry Ce6 through physical adsorption. PDA was then coated on the surfaces of the IrO_2_@MSN particles under alkaline conditions because DA self-polymerizes to form PDA. A BSA modifier was grafted onto the IrO_2_@MSN@PDA surface under ultrasonic conditions through an amidation reaction between the –COOH group of BSA and the –NH_2_ group of PDA in a buffered solution of Na_2_HPO_4_–NaH_2_PO_4_ to achieve colloidal stability (Scheme 1). The morphology of the product was analyzed using SEM and TEM. A spherical shape with an average diameter of approximately 84 ± 13 nm (Fig. 1a, b) was obtained. Therefore, we can conclude that NPs have a certain degree of aggregation, which is attributed to the interaction between the PDA and BSA that were grafted onto the NPs. The preparation of samples for SEM and TEM may have resulted in NP aggregation as well. The hydration kinetic diameter and Tyndall effect of the IrO_2_@MSN@PDA-BSA NPs in distilled water and PBS remained constant and clearly visible after 12 h of storage. This indicates that the surface-modified BSA endows durable colloidal stability to the IrO_2_@MSN@PDA-BSA NPs in different solutions. The difference in size of this nanocomposite between TEM and DLS may be due to the fact that the size measured by DLS is the hydration kinetic diameter: the BSA leads to the formation of a swollen structure with a high water content. The absence of vascular supportive tissues in tumors results in the formation of leaky vessels with pores (200 nm to 1.2 μm in diameter) and leads to poor lymphatic drainage, which is the structural basis of the enhanced permeability and retention (EPR) effect. The EPR effect of the nanomedicine with larger size was lower than the most suitable size but it also can accumulate in tumor through EPR effect. EDS (Additional file 1: Fig. S2) and elemental mapping (Fig. 1d–f) confirmed the coexistence of O, Si, and Ir. Nitrogen adsorption–desorption isotherms were used to investigate the porous structure (Fig. 1g). The results indicated that the IrO_2_@MSN@PDA-BSA NPs had a high specific surface area (149.6521 m²/g) and large pore volume (0.2 cm^3^/g). The average pore size was 5.7 nm (Fig. 1h). The XRD results of the IrO_2_@MSN@PDA-BSA NPs (Fig. 1i) did not contain typical diffraction peaks, indicating the poor crystalline state of the NPs. The chemical structure of IrO_2_@MSN@PDA-BSA NPs was determined through FTIR. The absorption peak at 1089 cm^− 1^ represents the tensile and asymmetric vibration of Si-O-Si (Additional file 1: Fig. S3), indicating the existence of SiO_2_ in the IrO_2_@MSN@PDA-BSA NPs. The vibration at 1422 cm^− 1^ is attributed to the –COOH group of BSA. The vibration signals at 1640 cm^− 1^ and 1500 cm^− 1^ are attributed to the deformation vibrations of amide I (–NH_2_) and amide II (–NH–) of BSA, respectively. The vibration peaks of the PDA and BSA amide groups due to the stretching of the –NH– group are located at 3400 cm^− 1^ and 3000 cm^− 1^, respectively. The peaks corresponding to the –C=C– stretching vibration of PDA is located at 1500 cm^− 1^. These results collectively confirm that PDA and BSA were successfully decorated onto the IrO_2_@MSN surface. The yields of IrO_2_-PVP, IrO_2_@MSN, IrO_2_@MSN@PDA-BSA, IrO_2_@MSN@PDA-BSA were 89.3%, 78.6%, 73.2 and 68.4%, respectively.

### Ce6 loading

**Fig. 2:**
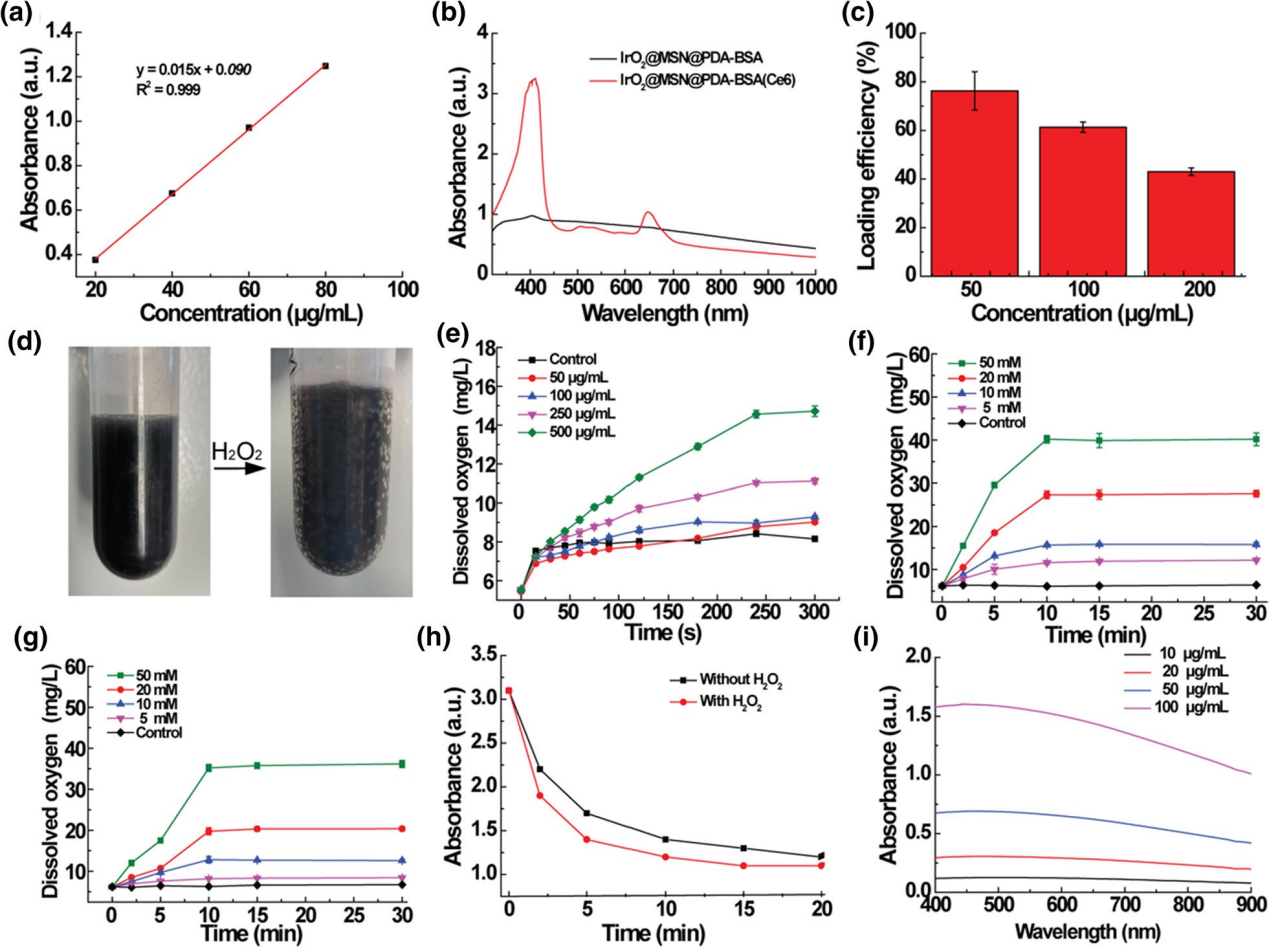
**a** Standard curve of Ce6; **b** light absorption of IrO_2_@MSN@PDA-BSA and IrO_2_@MSN@PDA-BSA(Ce6); **c** Ce6 loading efficiency of IrO_2_@MSN@PDA-BSA (1000 µg/mL) under different Ce6 concentrations; **d** photographs of IrO_2_@MSN@PDA-BSA solutions before and after the addition of H_2_O_2_; **e** O_2_ generation ability of IrO_2_@MSN@PDA-BSA with different concentrations; **f**, **g** O_2_ generation ability of IrO_2_@MSN@PDA-BSA solutions at **f** 37° C and **g** 80° C with various concentrations of H_2_O_2_; **h** the light absorption of IrO_2_@MSN@PDA-BSA(Ce6) with or without the addition of H_2_O_2_; **i** UV–vis spectra of IrO_2_@MSN@PDA-BSA NPs solutions

The conspicuous pore sizes of the IrO_2_@MSN@PDA-BSA NPs are likely to facilitate guest molecule loading. A classical PSA named Ce6 was chosen as the model drug to be physically adsorbed in the mesoporous pore of MSN. It is essential to evaluate the loading performance of IrO_2_@MSN@PDA-BSA because the tumor PDT is dose-dependent. The UV–vis–NIR spectra of IrO_2_@MSN@PDA-BSA before and after the loading of Ce6 are shown in Fig. 2b. The NPs carrying Ce6 have two typical characteristic peaks at 404 nm and 665 nm, in comparison to the spectrum of the pure IrO_2_@MSN@PDA-BSA. This proves that Ce6 was successfully loaded onto IrO_2_@MSN@PDA-BSA. The Ce6 loading of IrO_2_@MSN@PDA-BSA was then optimized by varying the concentration of Ce6. Ce6 concentrations of 50, 100, and 200 µg/mL resulted in Ce6 loading values of approximately 76.3%, 61.3%, and 43.0% on IrO_2_@MSN@PDA-BSA, respectively. The Ce6 loading ratio was calculated by dividing the weight of the loaded Ce6 with the weight of IrO_2_@MSN@PDA-BSA, which was equal to 3.8%, 6.1%, and 8.6% at Ce6 concentrations of 50, 100, and 200 µg/mL, respectively (Fig. 2c). According to the abovementioned results, a Ce6 concentration of 200 µg/mL was selected for the subsequent experiments. The in vitro Ce6 release behavior of IrO_2_@MSN@PDA-BSA(Ce6) was thoroughly evaluated. As shown in Additional file 1: Fig.S4, the Ce6 release percentages were lower than 12% under the tested conditions (pH = 7.4, T = 37 °C), thereby guaranteeing the secure and effective use of Ce6.

### Catalytic efficiency and ^1^O_2_ generating capacity

IrO_2_@MSN@PDA-BSA can improve the PDT efficiency by catalyzing the decomposition of H_2_O_2_ to generate endogenous O_2_ due to the catalase-like activity of IrO_2_. The addition of H_2_O_2_ to the IrO_2_@MSN@PDA-BSA solution results in the immediate generation of several O_2_ bubbles, as shown in Fig. 2d. This intuitively confirms the catalytic ability of IrO_2_. Further, the catalytic efficiency of IrO_2_@MSN@PDA-BSA was concentration-dependent. An increase in the IrO_2_@MSN@PDA-BSA concentration led to an increase in the concentration of the dissolved O_2_ (Fig. 2e). In addition, we investigate the catalytic efficiency of IrO_2_@MSN@PDA-BSA under simulated TME (pH = 6.0, the concentration of H_2_O_2_ was about 500 µM) conditions. As shown in Additional file 1: Fig. S4, although the content of generated oxygen decreased with the decrease of the solution pH, there was still a significant difference as compared to PBS with H_2_O_2_ at pH 6.0. The IrO_2_@MSN@PDA-BSA NPs possess the required thermal stability and are superior to the traditional catalase, which is vulnerable to hyperthermia (Fig. 2g). The catalytic ability of IrO_2_@MSN@PDA-BSA was satisfactory at 37 °C and highly dependent on the substrate concentration (Fig. 2f). The catalytic ability of IrO_2_@MSN@PDA-BSA remained constant even after increasing the temperature to 80 °C.

The amount of ^1^O_2_ contained in the solution was measured through light radiation to verify whether the O_2_ generated by IrO_2_@MSN@PDA-BSA could be utilized to enhance the tumor PDT. The irradiation of the IrO_2_@MSN@PDA-BSA solution led to a reduction in the light absorption by the DPBF, thereby confirming the generation of ^1^O_2_ (^1^O_2_ can oxidize the probe of DPBF and catalyze its discoloring reaction). In addition, it was observed that the rate of reduction in the light absorption increased with the addition of H_2_O_2_ (Fig. 2 h). This confirms the ability of the IrO_2_@MSN@PDA-BSA NPs to enhance ^1^O_2_ generation in the presence of H_2_O_2_. The improved ^1^O_2_ generation indicates that IrO_2_@MSN@PDA-BSA can be used as an intelligent nanozyme system to facilitate tumor PDT in the TME.

### Photothermal conversion performance

**Fig. 3:**
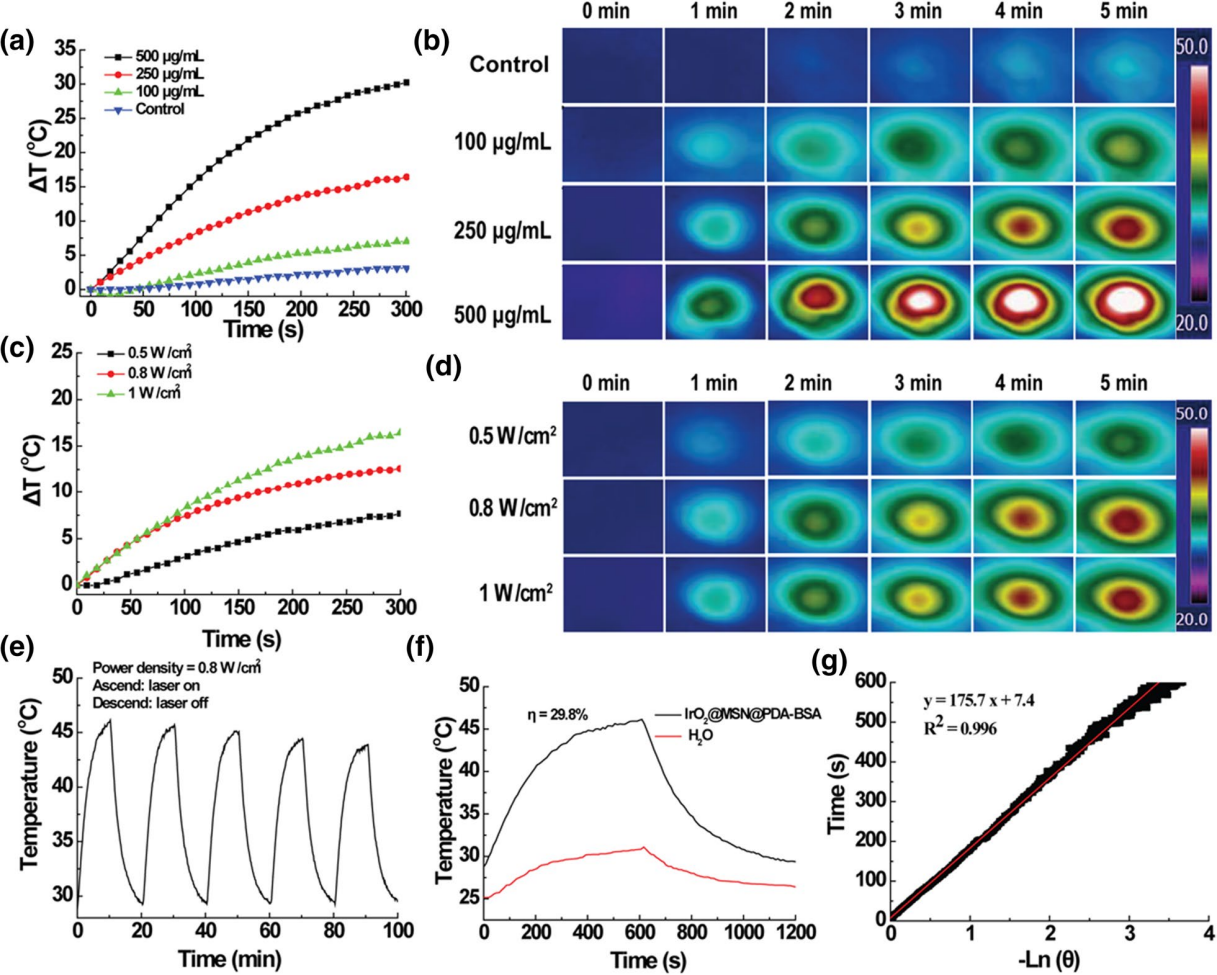
**a** Photothermal curves of IrO_2_@MSN@PDA-BSA NPs solution with different concentration upon 808 nm laser irradiation (5 min, 1 W/cm^2^); **b** the thermal images relevant to panel (**a**); **c** photothermal curves of IrO_2_@MSN@PDA-BSA NPs solution (250 mg/mL) under different laser power density irradiation (808 nm, 5 min); **d** the thermal images relevant to panel (**c**); **e** temperature curve of IrO_2_@MSN@PDA-BSA solution during 5 irradiation and cooling cycles (0.8 W/cm^2^); **f** steady-state heating curves of IrO_2_@MSN@PDA-BSA NPs and water; **g** time constant for heat transfer of IrO_2_@MSN@PDA-BSA NPs

In addition to enhancing the tumor PDT, IrO_2_@MSN@PDA-BSA is a PTA that can be used in the PTT of tumors. The absorbance capacity of IrO_2_@MSN@PDA-BSA for wavelengths ranging from 400 to 1000 nm was first studied. IrO_2_@MSN@PDA-BSA demonstrated a high light absorbance capacity, and the light absorbance increased with increasing NP concentration in the abovementioned wavelength range (Fig. 2i). The absorbed light was partially transformed into heat. The photothermal ability of IrO_2_@MSN@PDA-BSA was studied by varying the NIR laser power and NP concentration. The ΔT of IrO_2_@MSN@PDA-BSA increased with the increasing concentration of the solution at a fixed power density, as shown in Fig. 3a. However, the ΔT of water was negligible. The ΔT values of the solutions at concentrations of 100, 250, and 500 mg/mL were 7.0, 16.4, and 30.2 °C, respectively. IrO_2_@MSN@PDA-BSA was then used at a concentration of 250 mg/mL to study the impact of power density on heat production. The ΔT values of IrO_2_@MSN@PDA-BSA were 7.7, 12.6, and 16.4 °C at power densities of 0.5, 0.8, and 1.0 W/cm^2^ (808 nm), respectively. An E60 thermal imaging camera (FLIR Systems, Inc., USA) was used to record the concentration, irradiation, and laser-power-density-dependent temperature surges of IrO_2_@MSN@PDA-BSA (Fig. 3b, d). It was confirmed that the photothermal ability of IrO_2_@MSN@PDA-BSA was dependent on the material concentration and laser power density. The photothermal conversion efficiency and heat transfer time constant (τs) were equal to 29.8% and 175.7 s, both of which are comparable to the values obtained for graphene and black phosphorus nanosheets (Fig. 3f, g) [34, 35]. IrO_2_@MSN@PDA-BSA is considered photothermally stable because its ΔT variation during the five rounds of NIR laser irradiation substantial (Fig. 3e).

### In vitro biocompatibility assay

**Fig. 4:**
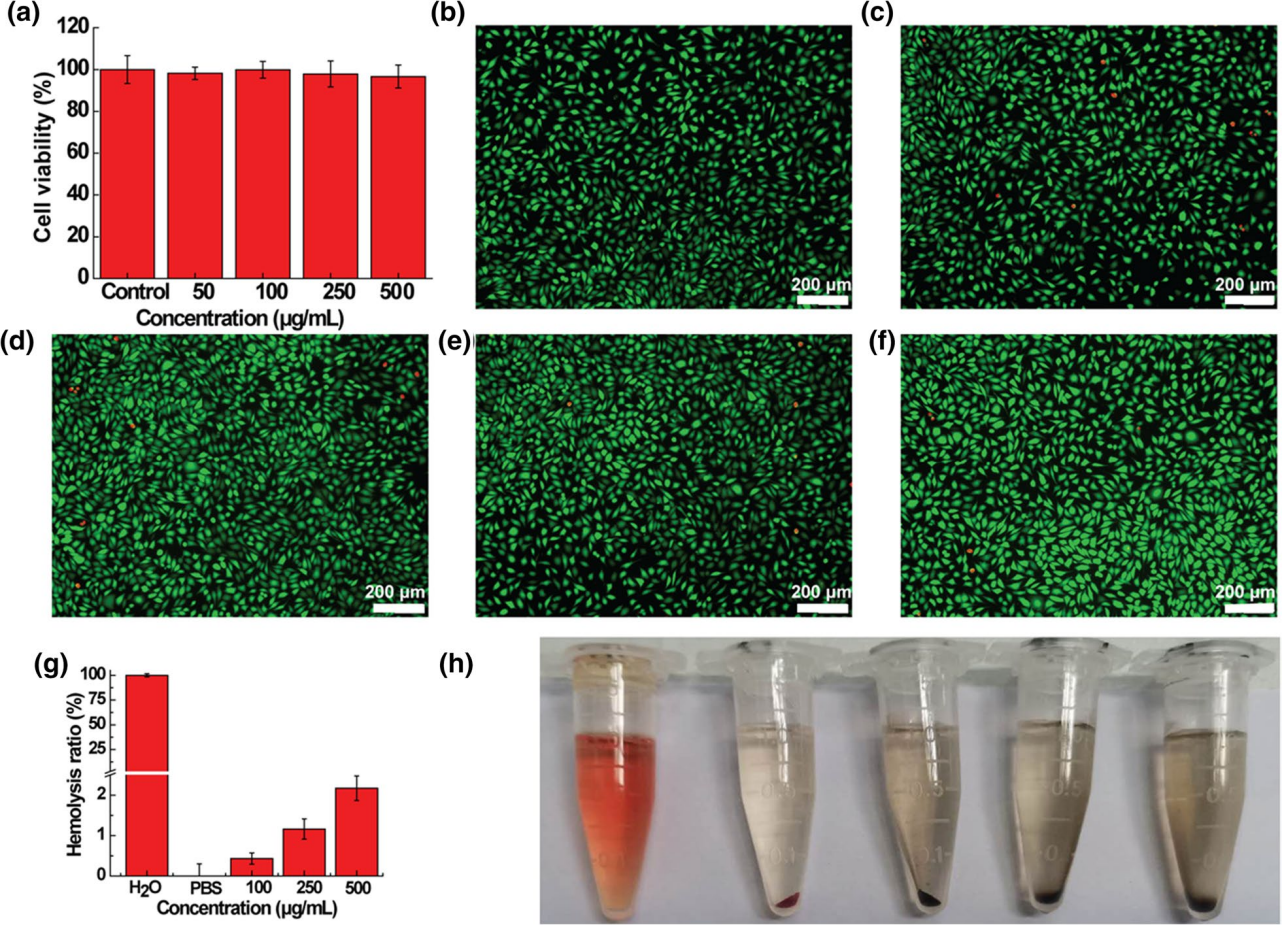
**a** L929 cells viabilities after cultured with different concentrations of IrO_2_@MSN@PDA-BSA(Ce6); **b**–**f** the photographs of Live/Dead staining relevant to (**a**); **g** hemolytic assay of IrO_2_@MSN@PDA-BSA(Ce6); **h** the photographic images relevant to (**g**)

In vitro biocompatibility, including cytocompatibility and hemocompatibility, is an essential factor in the biomedical applications of nanomaterials. Therefore, the cytocompatibility of IrO_2_@MSN@PDA-BSA(Ce6) was assessed using a CCK-8 cell viability assay and calcein-AM/PI double staining. The cell viabilities were maintained above 95% after 24 h of co-cultivation with varying concentrations of IrO_2_@MSN@PDA-BSA(Ce6), as shown in the CCK-8 results depicted in Fig. 4a. There were no evident differences between the abovementioned sample and the control group. These trends are similar to the results of CCK-8 and calcein-AM/PI double staining, which stained the live cells and dead cells green and red, respectively (Fig. 4b–f). This indicates that there were no obvious differences between the results of the experimental and control groups after culturing the samples for 24 h. These results indicate that the cytocompatibility of IrO_2_@MSN@PDA-BSA(Ce6) is satisfactory within experimental dosages. The hemocompatibility of IrO_2_@MSN@PDA-BSA(Ce6) was evaluated using a hemolysis assay. The HP of IrO_2_@MSN@PDA-BSA(Ce6) remained lower than 5%, even at a high material concentration of 500 µg/mL. The HP values were equal to 0.4 ± 0.14%, 1.2 ± 0.25%, and 2.16 ± 0.3% at IrO_2_@MSN@PDA-BSA(Ce6) concentrations of 100, 250, and 500 µg/mL, respectively (Fig. 4g). In addition, the mRBCs of the control and experimental groups were completely separated from the solution with relative ease, whereas the mRBCs in the positive control were completely ruptured (Fig. 4h). The hemolysis assay indicated that the NPs had a minimal impact on the structural integrity of mRBCs, thereby ensuring the safety of intravenous material injections.

### In vivo biocompatibility and biodistribution

**Fig. 5:**
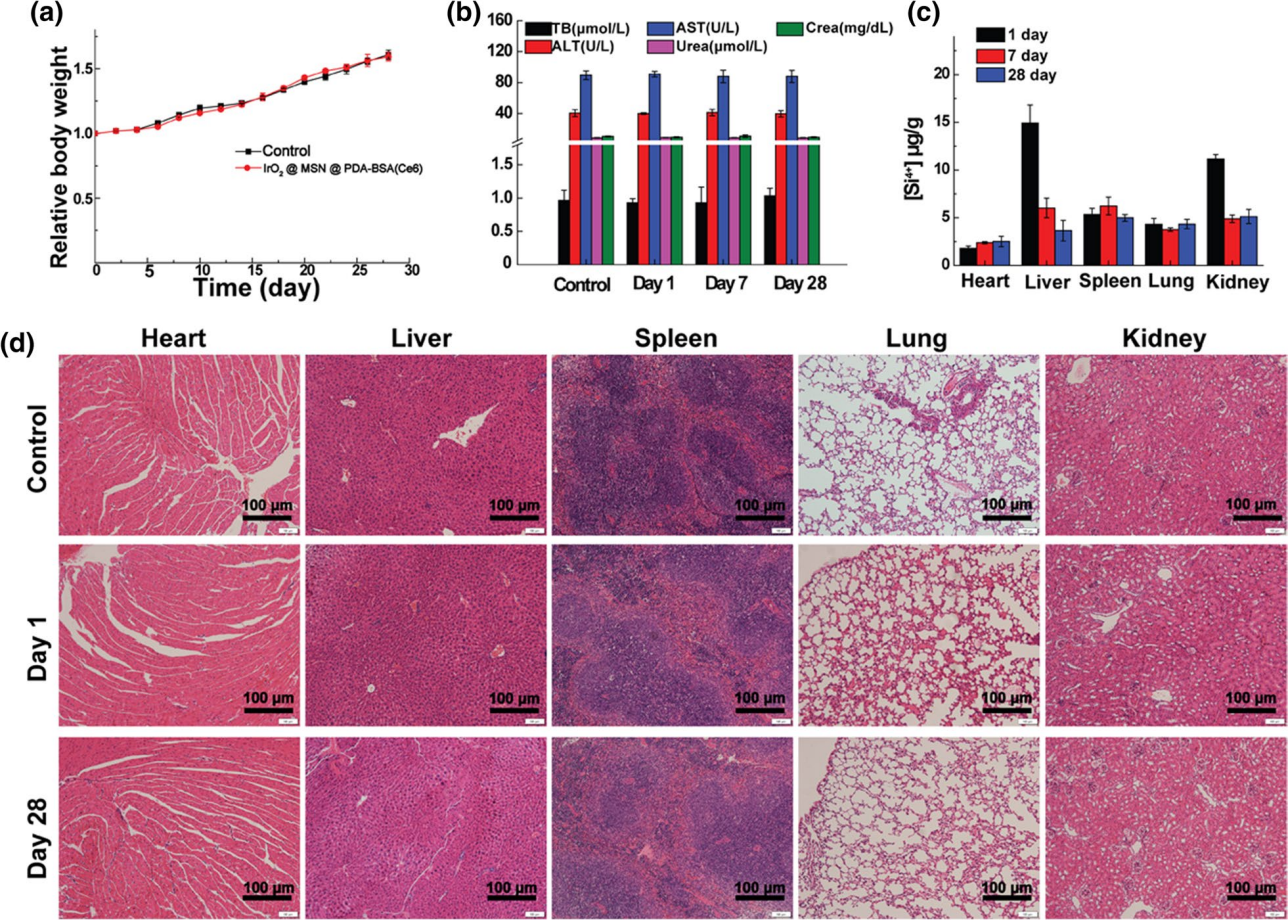
**a** Relative body weight of KM mice treated with saline or IrO_2_@MSN@PDA-BSA(Ce6) NPs (1 mg/mL); **b** blood biochemistry parameters; **c** biodistribution of Si ions in major organs of KM mice; **d** H&E staining of the major organs of KM mice treated with saline (control) or IrO_2_@MSN@PDA-BSA(Ce6) NPs

The biocompatibility of IrO_2_@MSN@PDA-BSA(Ce6) in animals was also studied. The body weights of mice were measured every 2 days after the I.V. materials injection. The body weights of the experimental and control groups did not differ significantly (p > 0.05), as shown in Fig. 5a. However, the blood routine (Additional file 1: Fig. S6) and serum biochemistry parameters (Fig. 5b) of the mice administered with IrO_2_@MSN@PDA-BSA(Ce6) were not similar to those of the control group (p > 0.05). The potential toxicity of IrO_2_@MSN@PDA-BSA(Ce6) to vital organs, such as the heart, liver, spleen, lungs, and kidneys, was also evaluated. The H&E staining of these organs demonstrated that no obvious damage was caused during the preset feeding periods (1, 7, and 28 days). This indicates that IrO_2_@MSN@PDA-BSA(Ce6) does not cause acute or chronic organ damage. The potential metabolic pathway of IrO_2_@MSN@PDA-BSA(Ce6) was then investigated by conducting an in vivo biodistribution of Si ions. The accumulation of Si in the liver and kidney 1 day after the injection was higher than that observed in the other organs due to the non-specific uptake of the reticuloendothelial system. The amount of Si in the major organs gradually decreased with time. As a result, the concentration of Si in the tested organs was lower than 5 µg/g 28 days after the injection.

### In vitro PTT/PDT tumor therapy

**Fig. 6:**
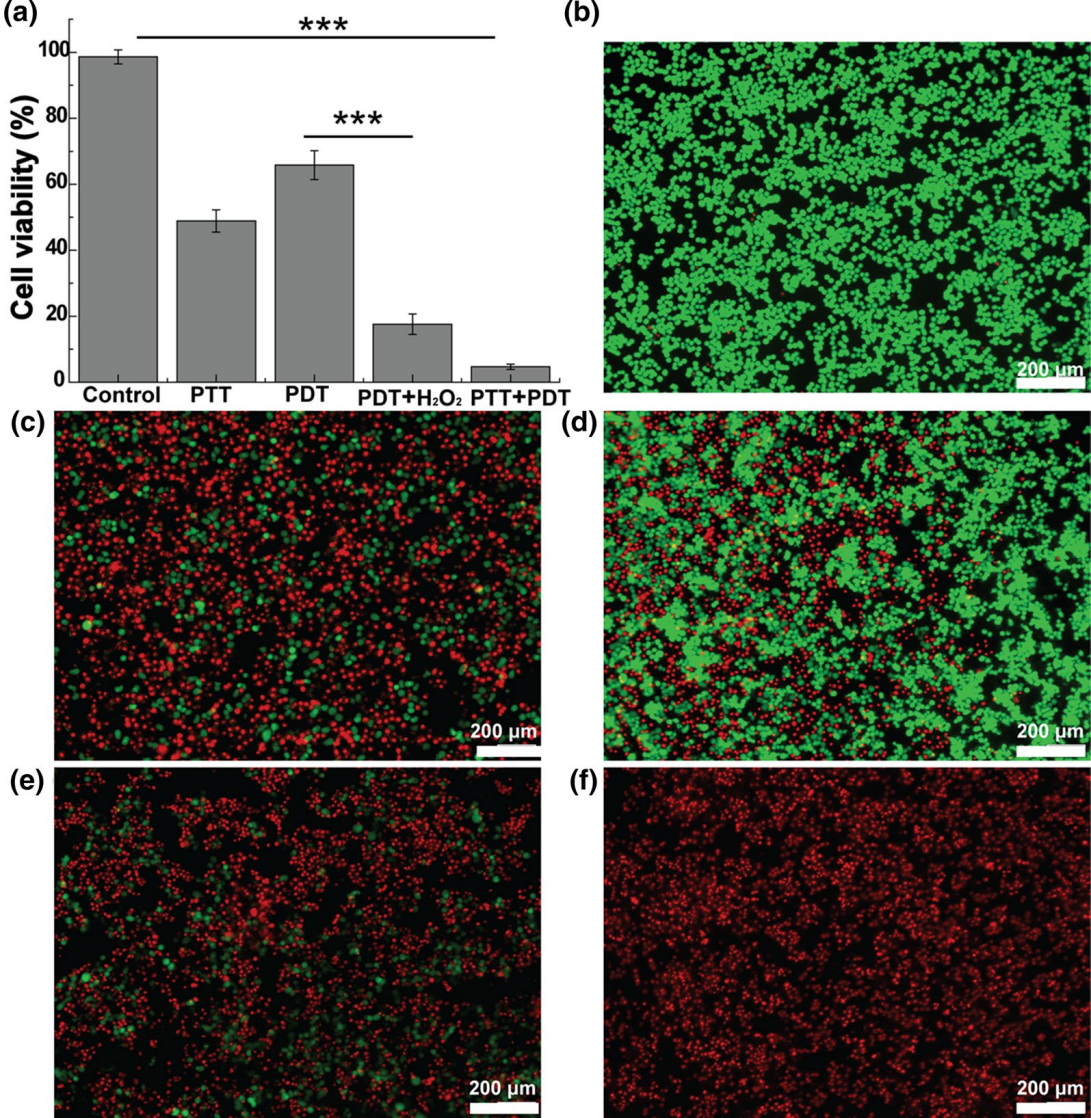
**a** HT29 cell viability after different treatments as noted; **b**–**f** the corresponding Live/Dead stained HT29 cells morphology (**b** control; **c** PTT (1 W/cm^2^, 5 min); **d** PDT (0.3 W/cm^2^, 3 min); **e** PDT + H_2_O_2_ (0.3 W/cm^2^, 3 min); **f** PTT + PDT (1 W/cm^2^, 5 min; 0.3 W/cm^2^, 3 min)

The efficiency of the combined tumor therapy in HT-29 cells and tumor-bearing nude mice was studied to realize the desirable biocompatibility of IrO_2_@MSN@PDA-BSA(Ce6) in vitro and in vivo. The HT-29 cells cultured with IrO_2_@MSN@PDA-BSA(Ce6) in the absence of light intervention did not report excessive cell death. However, the survival rate of the cells after being subjected to 808 nm NIR laser irradiation was reduced to 48.9% (***p < 0.001, versus control, Fig. 6a) due to the excellent photothermal conversion ability of IrO_2_@MSN@PDA-BSA(Ce6). In comparison with the control group, the viability of the HT-29 cells irradiated with a 660 nm laser decreased to 65.8% (PDT group, ***p < 0.001, versus control). The addition of H_2_O_2_ to the DMEM under conditions similar to those of the PDT group drastically reduced the viability of the HT-29 cells to 17.6% (***p < 0.001, versus control; ***p < 0.001, versus PDT). The differences in the cell viability values indicate that the co-existence of H_2_O_2_ and IrO_2_@MSN@PDA-BSA(Ce6) could enhance the tumor PDT effect. This is because IrO_2_ catalyzed the decomposition of H_2_O_2_ to produce endogenous oxygen, which was further sensitized by the Ce6 while being irradiated by a 660 nm laser. Owing to the PTT and CAT-mimicking abilities of IrO_2_@MSN@PDA-BSA(Ce6), the HT-29 cells were almost completely dead after being irradiated by the 808 nm and 660 nm lasers successively (***p < 0.001, versus control). The results of the calcein-AM/PI Live/Dead staining were similar to those of the CCK-8 cell viability assay. The HT-29 cells in the control group were stained with a strong green fluorescence, whereas most HT-29 cells treated with PTT, PDT, enhanced PDT, and PDT/PTT were stained red (Fig. 6b–f). This validates the in vitro therapeutic effect of IrO_2_@MSN@PDA-BSA(Ce6).

### In vivo combined tumor therapy

**Fig. 7:**
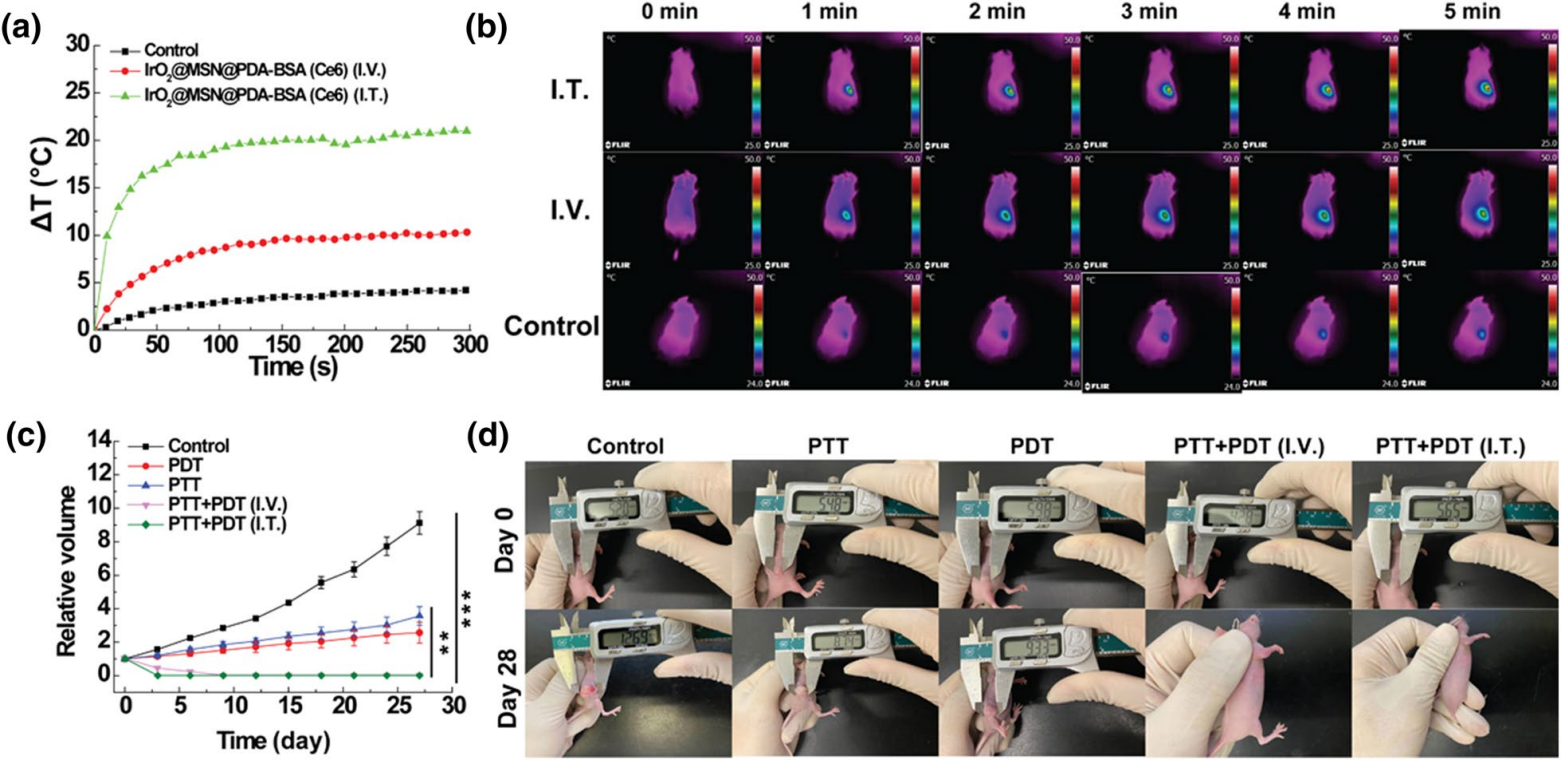
**a** The tumor temperature under NIR laser irradiation (PTT, 1 W/cm^2^, 5 min; PDT, 0.3 W/cm^2^, 3 min); **b** the corresponding thermal images of mice of panel (**a**); **c** relative tumor volume change curves of mice after different treatments as noted; **d** pictures of mice at days 0 and 28 of panel (**c**)

The in vitro therapeutic effect investigation provides preliminary confirmation that IrO_2_@MSN@PDA-BSA(Ce6) is suitable for multimodal tumor therapy. This was verified by the tests conducted on animals. The variations in the temperatures of the tumor-bearing nude mice that received the material injections were measured using a thermal imaging camera. The ΔT of the control group was only 4.3 °C after being subjected to NIR irradiation at 808 mm (5 min), whereas ΔT for the mice injected with the I.V. and I.T. materials were 10.3 and 20.8 °C, respectively. The variation in the temperatures of the mice injected with the I.V. material also proved that IrO_2_@MSN@PDA-BSA(Ce6) could passively accumulate at the tumor site through the EPR effect. The volume of the tumors of the mice belonging to the control group increased to 9.12 times their original values after 28 days of feeding. However, the tumor volumes of the PTT group increased to 2.56 times their original values (***p < 0.001, versus control). This indicates that PTT can kill a few cancer cells while the surviving ones continue to grow. Similarly, the tumor volumes of the PDT group increased to approximately 3.42 times their original values (***p < 0.001, versus control). There was no obvious difference between the tumor volumes of the PDT and PTT groups (p > 0.05). This suggests that a single dose of PTT or PDT produces a certain therapeutic effect. However, the tumors of the mice in the combined therapy group were completely eradicated, irrespective of the material injection method (Fig. 7c, d). This confirms the superior efficiency of the combined tumor therapy of the IrO_2_@MSN@PDA-BSA(Ce6) NPs.

## Conclusions

An IrO_2_@MSN@PDA-BSA(Ce6) nanoenzymatic composite platform for the treatment of tumors through the combined effects of PTT and PDT was designed in this study. The MSN coating introduced abundant pores for Ce6 loading, and the conjugated BSA endowed the NPs with sufficient biocompatibility in vitro and in vivo. The loading of Ce6 facilitated the photo-triggered generation of single oxygen, resulting in photodynamic cell death. IrO_2_ functions as a CAT-mimic to catalyze the decomposition of H_2_O_2_ in the TME to generate endogenous oxygen and alleviate the hypoxia of solid tumors. This enhances the PDT of Ce6 by offering local oxygen and promoting the generation of ^1^O_2_. In addition, the PDA coating and IrO_2_ NPs exhibited remarkably high photothermal conversion efficiencies, resulting in the ablation of solid tumors through hyperthermia. The therapeutic effect of IrO_2_ @MSN@PDA-BSA(Ce6) for multimodal tumor therapy was proved both in vitro and in vivo. The results indicated that the tumors of the mice in the combined therapy group could be completely eradicated. The findings of the present study highlight the feasibility of using theranostic nanoenzymes for translational medicine.

## Supplementary Information

**Additional file 1.** 1. Materials. 2. Characterization of IrO_2_@MSN@PDA-BSA NPs. **Fig. S1.** (a) The TEM images of IrO_2_@MSN@PDA-BSA NPs; (b) the corresponding size distributions of IrO_2_-PVP nanoparticles. **Fig. S2.** EDS spectra of IrO_2_@MSN@PDA-BSA NPs. **Fig. S3.** FTIR spectra of PVP, PDA, and IrO_2_@MSN@PDA-BSA NPs. **Fig. S4.** Release of Ce6 from IrO_2_@MSN@PDA-BSA(Ce6) NPs. **Fig. S5.** Variation of the dissolved oxygen (DO) content at different pH values (pH 6.0 and 7.4). **Fig. S6.** Routine blood test of IrO_2_@MSN@PDA-BSA(Ce6) treated KM mice fed for different days.

## Data Availability

All data generated or analyzed during this study are included in this manuscript and its additional material.
