# Supplementary material for: Synthesis of iridium-based nanocomposite with catalase activity for cancer phototherapy
Source: J Nanobiotechnology. 2021 Jul 7;19:203. doi: 10.1186/s12951-021-00948-8 (PMC8265148; doi:10.1186/s12951-021-00948-8)
Supplement: Supplementary file 1 — Additional file 1. 1. Materials. 2. Characterization of IrO2@MSN@PDA-BSA NPs. Fig. S1. (a) The TEM images of IrO2@MSN@PDA-BSA NPs; (b) the corresponding size distributions of IrO2-PVP nanoparticles. Fig. S2. EDS spectra of IrO2@MSN@PDA-BSA NPs. Fig. S3. FTIR spectra of PVP, PDA, and IrO2@MSN@PDA-BSA NPs. Fig. S4. Release of Ce6 from IrO2@MSN@PDA-BSA(Ce6) NPs. Fig. S5. Variation of the dissolved oxygen (DO) content at different pH values (pH 6.0 and 7.4). Fig. S6. Routine blood test of IrO2@MSN@PDA-BSA(Ce6) treated KM mice fed for different days. [file 12951_2021_948_MOESM1_ESM.docx]

**Additional Information**

**Synthesis of Iridium-Based Nanocomposite with Catalase Activity for Cancer Phototherapy**

Hang Wu,^a,1^ Qi Jiang,^b,1^ Keyi Luo,^c^ Chunping Zhu,^b^ Mengmeng Xie,^c^ Shige Wang,^c^ Zhewei Fei^a*^,and Jiulong Zhao,^b*^

^a^ Department of Breast Surgery, Xinhua Hospital, Shanghai Jiaotong University School of Medicine, No. 1665 Kongjiang Road, Shanghai 200433, People's Republic of China

^b^ Department of Gastroenterology, Changhai Hospital, Second Military Medical University, No. 168 Changhai Road, Shanghai 200433, People's Republic of China

^c^ College of Science, University of Shanghai for Science and Technology, No. 334 Jungong Road, Shanghai 200093, People's Republic of China

^1^ These authors contributed equally to this work. ^*^To whom correspondence should be addressed email: zheweifei2013@sina.cn (Prof. Fei), jlzhao9@163.com (Dr. Zhao)

**1.Materials**

Iridium chloride (IrCl_3_), polyvinylpyrrolidone (PVP), sodium hydroxide (NaOH), hexadecyl trimethyl ammonium bromide (CTAB), tetraethyl orthosilicate (TEOS), ethanol, ammonia solution (NH_3_·H_2_O), dopamine hydrochloride (DA), and 1,3-diphenylisobenzofuran (DPBF) were commercially obtained from Aladdin Bio-Chem Technology Co., Ltd. (Shanghai, China). BSA (fraction V) was purchased from Sangon Biotech Co., Ltd. (Shanghai, China). Mice fibroblast cells (L929) and human colorectal carcinoma (HT29) cells were obtained from the Institute of Biochemistry and Cell Biology, which be owned by the Chinese Academy of Sciences (Shanghai, China). Dulbecco's Modified Eagle Medium (DMEM), phosphate buffer saline (PBS), and Roswell Park Memorial Institute-1640 medium (RPMI-1640) were procured from Corning Co., Ltd. (Shanghai, China). L929 was cultured in RMPI-1640 and HT29 was cultured in DMEM supplemented with 10% fetal bovine serum (FBS, Gibco, Shanghai, China), 100 U/mL penicillin, and 100 μg/mL streptomycin (Gibco, Shanghai, China) in humidified at 37°C under 5% CO_2_. Cell culture flasks and plates were purchased from Corning Co., Ltd. (Shanghai, China). The Cell counting kit-8 (CCK-8) was purchased from Dojindo Laboratories (Japan). Balb/c nude mice and Kunming (KM) mice (female, 4-6 weeks, 20-25 g) were ordered from Shanghai Slac Laboratory Animal Center (Shanghai, China). All experimental mice are bred strictly in accordance with the rules and regulations of the Minister of Health of the People's Republic of China(MOHC). All water used in this research was purified by the Milli-Q Plus 185 water purification system (Millipore, Bedford, MA) to achieve a resistivity higher than 18.2 MΩ·cm. Every chemical was used after being gained.

**2. Characterization of IrO_2_@MSN@PDA-BSA NPs**

The particle size, morphology and X-ray energy dispersive spectroscopy (EDS) of IrO_2_@MSN@PDA-BSA was analyzed with scan electron microscopy (SEM, SIGMA). The particle morphology was also monitored using the transmission electron microscopy (TEM, JEOL-2100F analytical electron microscope). We used Micromeritics Tristar II 3020 (Micromeritics Instrument Corporation, USA) to conducted Nitrogen adsorption–desorption isotherm measurements at 77 K. The Uv-vis-NIR spectrophotometer of IrO_2_@MSN@PDA-BSA was recorded by a Uv-vis-NIR spectrophotometer (Lambda 25, PerkinElmer, USA). The chemical information of the IrO_2_@MSN@PDA-BSA was measured by a Fourier transform infrared spectroscopy (FTIR, Nicolet 7000-C spectrometer). We used Rigaku D/max-2200 PC X-ray diffraction (XRD) system to analyze the crystalline structure of the materials. The hydrodynamic size of IrO_2_@MSN@PDA-BSA in DI water and PBS was characterized by a dynamic light scattering instrument (DLS, Nano ZS 90, Malvern, UK).

**3. Additional figures**

**
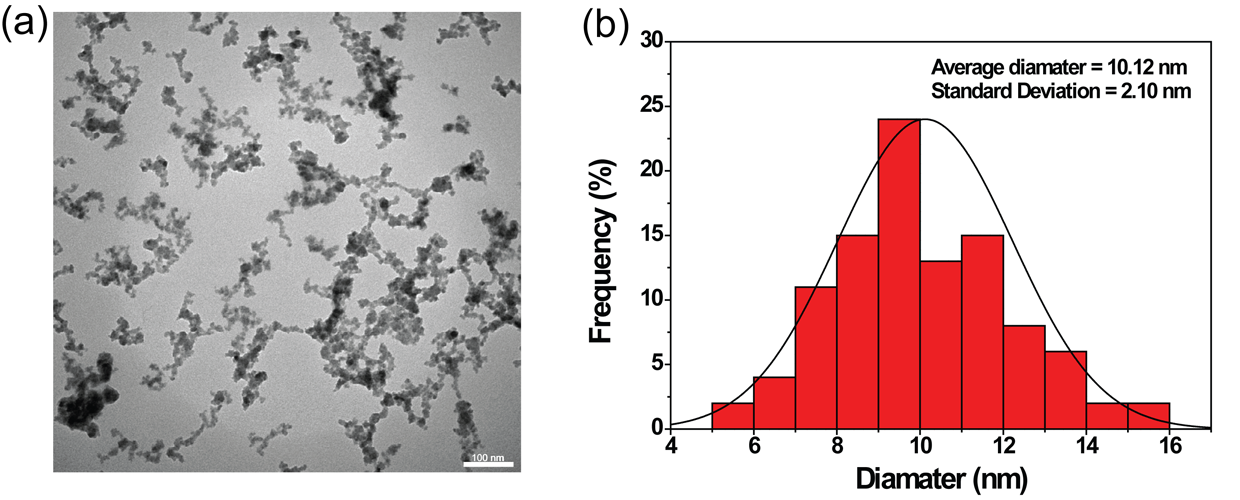
**

**Fig S1.** (a) The TEM images of IrO_2_-PVP nanoparticles; (b) the corresponding size distributions of IrO_2_-PVP nanoparticles.





**Fig S2.** EDS spectra of IrO_2_@MSN@PDA-BSA NPs





**Fig S3.** FTIR spectra of PVP, PDA, and IrO_2_@MSN@PDA-BSA NPs


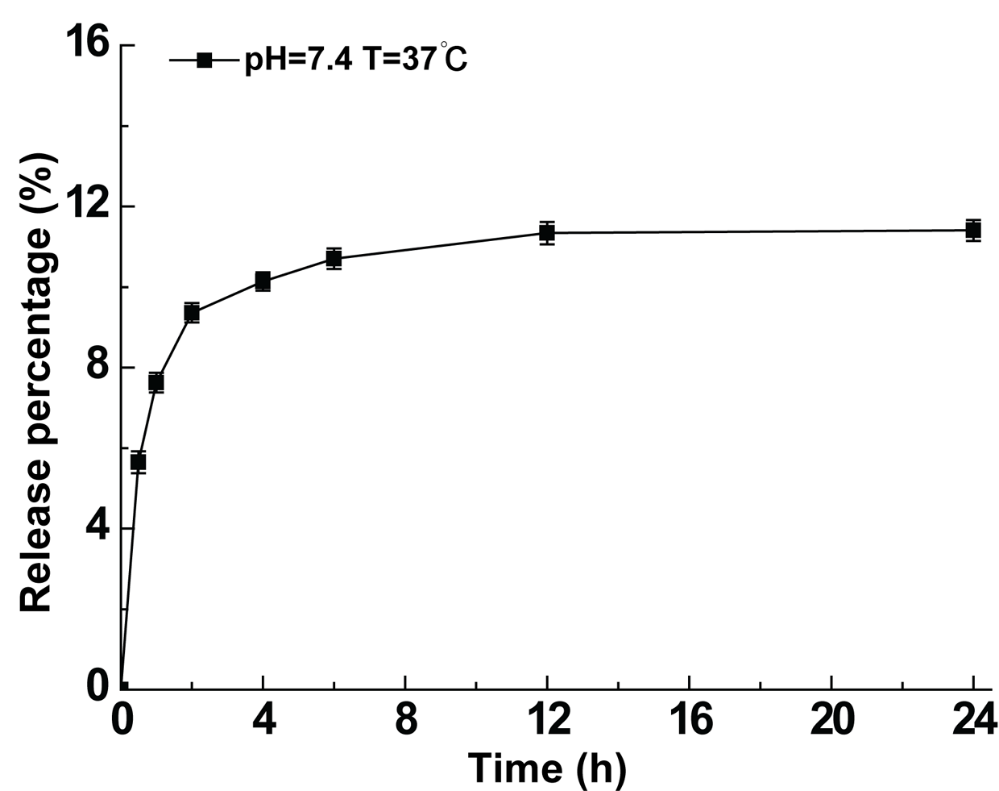


**Fig S4.** Release of Ce6 from IrO_2_@MSN@PDA-BSA(Ce6) NPs


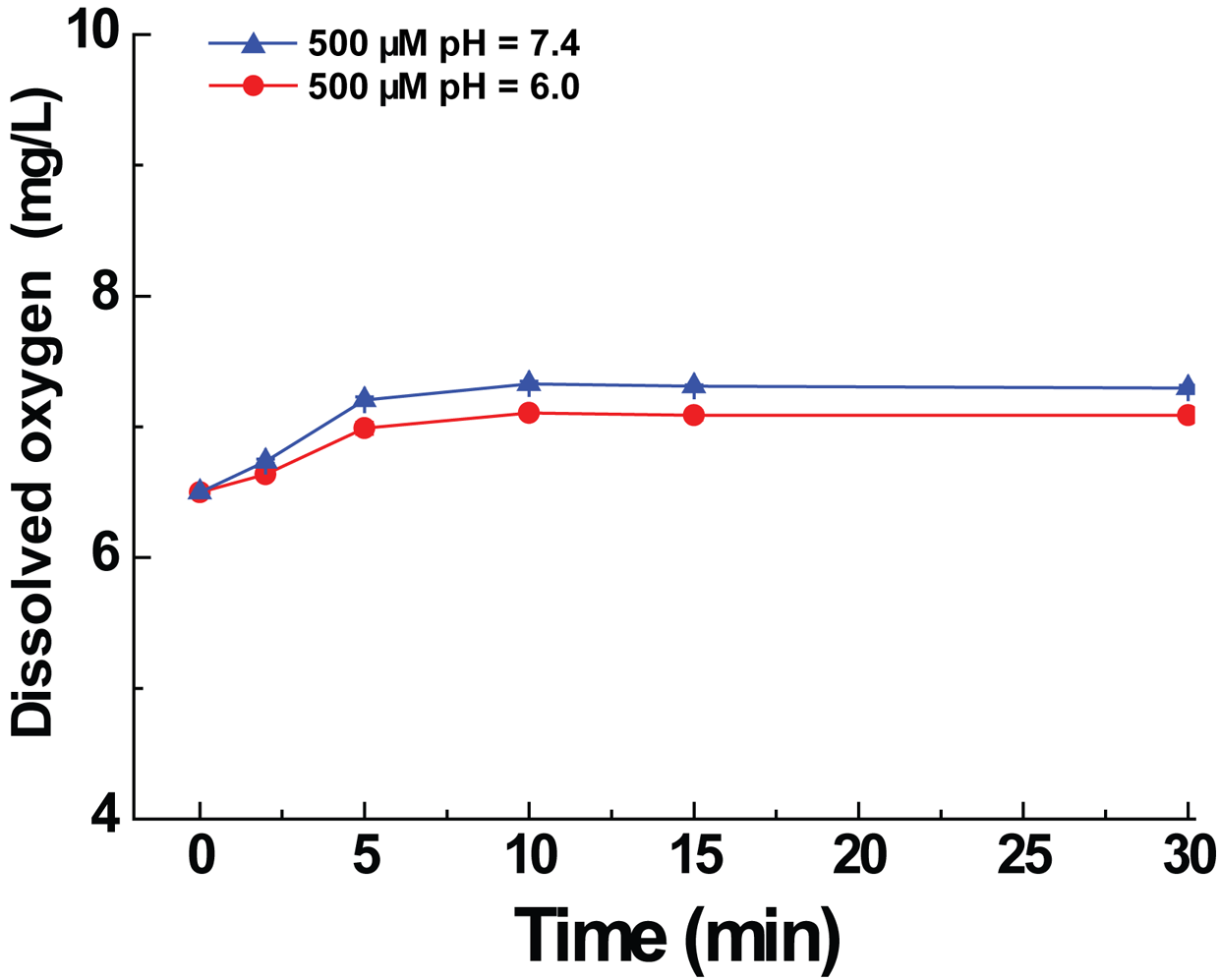


**Fig S5.** Variation of the dissolved oxygen (DO) content at different pH values (pH 6.0 and 7.4)

**
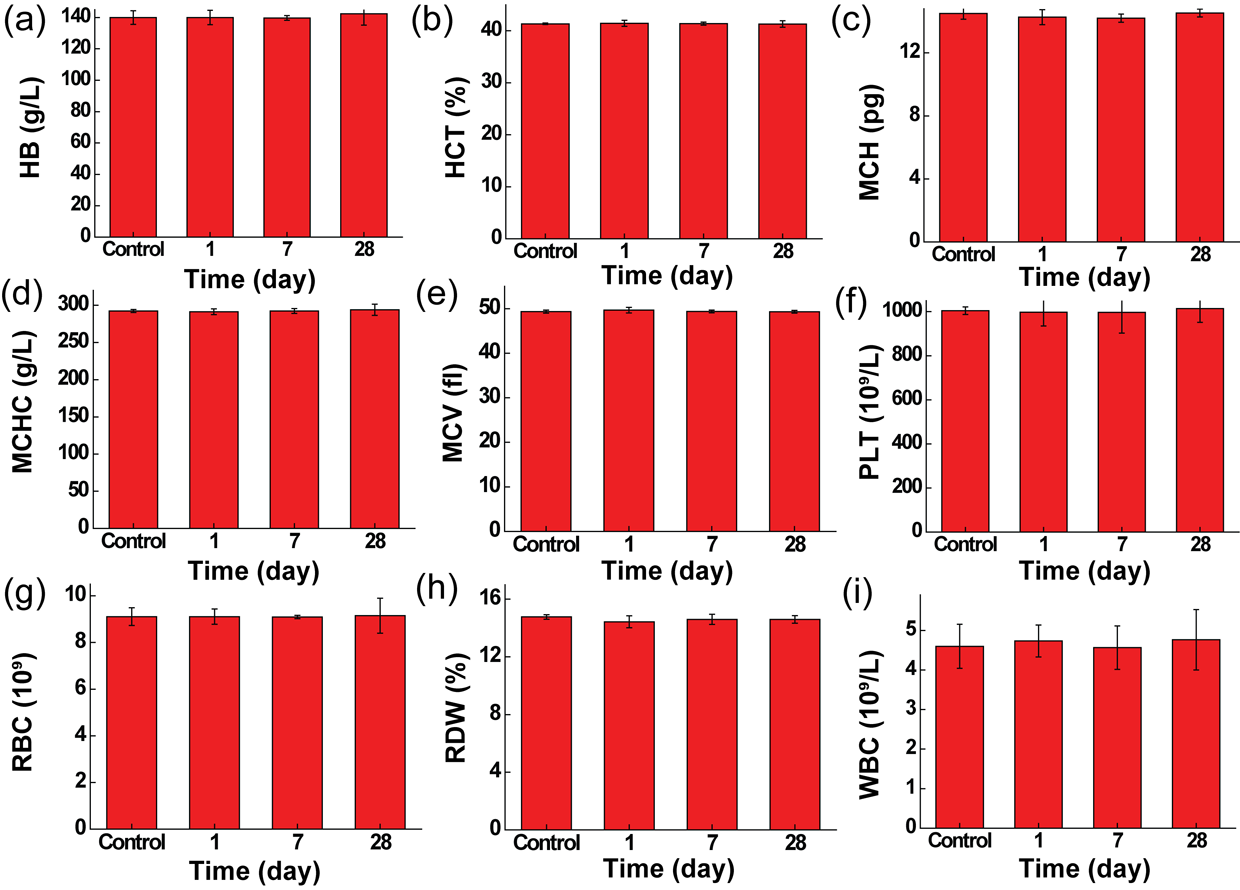
**

**Figure S6.** Routine blood test of IrO_2_@MSN@PDA-BSA(Ce6) treated KM mice fed for different days. (a) Hemoglobin (HGB); (b) hematocrit (HCT);(c) mean corpuscular hemoglobin (MCH); (d) mean corpuscular hemoglobin concentration (MCHC); (e) mean corpuscular volume (MCV); (f) platelet (PLT); (g) red blood cell count (RBC); (h) red cell distribution width (RDW); (i) white blood cell count (WBC).
